# Detection of duck adenovirus 3 using RAA-CRISPR/Cas12a based lateral flow dipstick method

**DOI:** 10.3389/fmicb.2026.1815110

**Published:** 2026-05-05

**Authors:** Wenyu Zhang, Zhiwang Tang, Siwen He, Luoye Wang, Yongshan Zhou, Tingting Lu, Shuyu Chen, Yu Huang, Chunhua Zhu, Chunhe Wan, Wensong Jin, Jiayu Li

**Affiliations:** 1 School of Life Sciences, Fujian Agriculture and Forestry University, Fuzhou, China; 2 Institute of Animal Husbandry and Veterinary Medicine/Fujian Key Laboratory for Avian Diseases Control and Prevention, Fujian Academy of Agricultural Sciences, Fuzhou, China

**Keywords:** CRISPR/Cas12a, detection, duck adenovirus 3, lateral flow dipstick, recombinase aided amplification

## Abstract

Duck adenovirus 3 (DAdV-3) was firstly identified in Muscovy ducklings exhibiting slight pericardial effusion, swelling, and hemorrhaging in the kidneys, which is a poorly characterized duck virus. This study established a CRISPR/Cas12a coupled with a Lateral flow dipstick (RAA-CRISPR/Cas12a-LFD) detection platform for DAdV-3. The core mechanism of this platform involves a three-step cascade reaction pathway. First, the target sequence of the DAdV-3100 k gene is amplified via RAA isothermal amplification. Second, the amplified products activate the trans-cleavage activity of Cas12a. Finally, signal output is achieved by cleaving probes on the Lateral flow dipstick. This method exhibits high sensitivity, with a minimum detection limit of 2.87 × 10^2^ copies/μL for the DAdV-3 positive plasmid. It also demonstrates high specificity for DAdV-3, showing no cross-reactivity with other common duck-origin viruses such as avian influenza virus (AIV), Newcastle disease virus (NDV), duck Tembusu virus (DTMUV), duck plague virus (DPV), Muscovy duck parvovirus (MDPV), duck hepatitis A virus type 1 (DHAV-1), and duck reovirus (DRV). The method enables on-site detection of DAdV-3 within 1 h and 20 min. It does not rely on standardized molecular laboratories or professional operation, provides visualized detection results, allows rapid detection at the grassroots level, and has low detection costs, making it suitable for promotion in small-scale farms. This approach provides a technical means for the detection and epidemiological investigation of DAdV-3.

## 1 Introduction

Since its first discovery and isolation from Muscovy ducks with liver hemorrhage and necrosis in Guangdong, China in 2014 (Zhang et al., 2016), DAdV-3 has seen an expanding host range and increasing incidence. Related cases have been reported in Fujian (Chen et al., 2019a), Jiangsu (Lin et al., 2025), and Anhui (Yin et al., 2022) provinces in Southern China. The disease is widespread among waterfowl, causing particularly severe economic impacts on the duck farming industry. Clinical symptoms include swelling and hemorrhage of the liver and kidneys, mottled or scattered punctate hemorrhages on the liver surface, a yellowish-white liver, and mild hepatitis-hydropericardium syndrome (HHS) (Tang et al., 2025).

The International Committee on Taxonomy of Viruses (ICTV) classifies duck adenoviruses into two categories: Duck Atadenovirus A (DAdV-A), which belongs to the genus Atadenovirus, and Duck Aviadenovirus B (DAdV-B), which belongs to the genus Aviadenovirus (Shi et al., 2019). Although DAdV-3 exhibits certain differences from DAdV-B in terms of nucleotide homology and viral structure, it is considered to belong to the Aviadenovirus genus due to its basic structure resembling that of typical aviadenoviruses. However, the name for this virus has not yet been officially recognized by the ICTV. DAdV-3 virus exhibits typical adenovirus morphology; virions are approximately 70 nm in diameter, spherical, non-enveloped, and possess an icosahedral symmetry structure (Zhang et al., 2024). The virus replicates within the nucleus, and virions are arranged in crystalline arrays. Hexon is the most abundant capsid protein in adenoviruses and serves as the major surface antigen; it forms the 240 facets of the icosahedral capsid as trimers. 100 K is a specific non-structural protein of adenovirus; during the late phase of infection, the 100 K protein is synthesized in large amounts in the cytoplasm. It specifically recognizes and binds to newly synthesized hexon monomers, assists in their proper folding, and promotes the assembly of three monomers into a stable trimeric structure. Furthermore, the 100 K protein remains associated with the newly formed trimers and, utilizing its nuclear localization signal (NLS), guides the entire hexon-100 K complex into the nucleus to facilitate the formation of new virus particles. This effectively increases the efficiency of viral protein synthesis and consequently inhibits host protein synthesis. Moreover, the 100 K gene is specific to adenoviruses, and its sequence is relatively conserved with critical functions; therefore, this study is based on the 100 K gene (Hong et al., 2025; Koyuncu et al., 2013; Shah et al., 2016).

Diagnostic methods for aviadenoviruses include serological and molecular approaches (Cheng et al., 2020; Zhou, 2023). Enzyme-linked immunosorbent assay (ELISA) is widely employed due to its high specificity, sensitivity, and reproducibility. Recombinant conserved structural proteins (Fiber-1 and Fiber-2) and nonstructural proteins (100 K and 33 K) have been expressed in prokaryotic systems and used as coating antigens to develop indirect ELISAs for DAdV-3 antibodies (Zhang, 2023; Chen et al., 2019b). However, these serological assays require high-quality antigen preparations and skilled technical operation, limiting their utility for large-scale screening. Molecular diagnostics, mainly include polymerase chain reaction (PCR) (Wan et al., 2019), real-time quantitative PCR (qPCR), multiplex PCR, and loop-mediated isothermal amplification (LAMP) (Wan et al., 2018; Zhou et al., 2024; Xie et al., 2011). Although PCR is sensitive and simple, it depends on precise thermal cycling and advanced instrumentation, constraining its applicability in resource-limited, field settings. Since most duck farms are small and lack diagnostic infrastructure, there is a critical need for rapid, simple, cost-effective, and field-deployable assays for surveillance and outbreak control.

Recombinase-aided amplification (RAA) is an isothermal nucleic acid amplification method that exploits the coordinated action of recombinase, single-stranded DNA-binding protein, and DNA polymerase to rapidly amplify target sequences at constant temperature (37–42 °C) with high specificity and operational simplicity (Xie et al., 2026; Chen W. et al., 2025). Compared to conventional PCR, RAA requires shorter incubation time and less stringent temperature control, reducing equipment dependency and overall cost. The clustered regularly interspaced short palindromic repeats (CRISPR)/Cas12a system enables crRNA-guided specific recognition of target DNA, triggering trans-cleavage activity that amplifies signal output with high sensitivity and specificity when combined with fluorescent reporters or Lateral flow dipstick (LFD) for visual readout (Koonin et al., 2017; Zhi et al., 2022; Zheng et al., 2025). In this study, we developed a rapid RAA-CRISPR/Cas12a-LFD assay targeting the 100 K gene for specific detection of DAdV-3. This method provides a simple, rapid, and field-applicable diagnostic tool suitable for primary-level surveillance and control of DAdV-3.

## 2 Materials and methods

### 2.1 Plasmid DNA, virus samples and sample nucleic acid extraction

The 100 K gene plasmid of DAdV-3 was synthesized by Sangon Biotech (Shanghai, China) and diluted to 2.87 × 10^9^ copies/μL as a standard reference. Positive nucleic acid samples of DAdV-3, avian influenza virus (AIV), Newcastle disease virus (NDV), Duck Tembusu virus (DTMUV), Duck plague virus (DPV), Muscovy duck parvovirus (MDPV), Duck hepatitis A virus type 1 (DHAV-1), and Muscovy duck reovirus (DRV) were obtained and authenticated by the Fujian Provincial Institute of Animal Husbandry and Veterinary Medicine. Viral nucleic acid extraction was performed using the YALEPIC® Viral DNA/RNA Isolation Kit from Yali Biotech (Jiangsu, China). This kit offers high extraction efficiency and broad applicability, enabling efficient extraction from various clinical samples. The extracted nucleic acid can be directly used for PCR amplification or stored at −20 °C.

### 2.2 Reagents

RAA amplification reagents, CRISPR/Cas12a reaction components (lateral flow format), and nucleic acid test dipstick (for CRISPR SHERLOCK) were procured from Shenzhen Yizhi Biotech (Guangdong, China). The DL500 DNA Marker was supplied by Takara Bio (Dalian, China). Nucleic acid stain was obtained from Uelandy Biotechnology (Suzhou, China), and agarose was sourced from Beyotime Biotechnology (Shanghai, China). Other routine chemicals and consumables were purchased from Sangon Biotech (Shanghai, China).

### 2.3 Design and synthesis of RAA primers and crRNA

The gene sequence of DAdV-3 was downloaded from GenBank database,^1^ and conserved regions within the 100 K gene were selected as targets. Based on established principles for RAA primer and crRNA design, five pairs of specific RAA primers targeting the DAdV-3100 K gene (DAdV-3-100 k-F1/R1, DAdV-3-100 k-F2/R2, DAdV-3-100 k-F2/R3, DAdV-3-100 k-F3/R1, DAdV-3-100 k-F4/R4) and DAdV-3-100 k-crRNA were designed (Table 1). All primers and crRNAs were synthesized by Shenzhen Yizhi Biotech (Guangdong, China).

**Table 1:** Primer and crRNA sequences.

Primers	Sequences (5′–3′)	Position
DAdV-3-100k-F1	TGCCATATAGACTTGGGGAAAGACTTCTCCA	121–151
DAdV-3-100k-F2	CGGAAGAAGAGGTATTAGGCATGGGAGACGC	230–260
DAdV-3-100k-F3	CGGATGCCATATAGACTTGGGGAAAGACTTC	117–147
DAdV-3-100k-F4	CAGAGCGGAAGAAGAGGTATTAGGCATGGGA	225–255
DAdV-3-100k-R1	TCATTCTCGTCACTGACACTCGACTCAGTGT	329–359
DAdV-3-100k-R2	CCTCCAGTGTTTCGGTCTCGGTGGAGACCGG	412–442
DAdV-3-100k-R3	CGCTCAGCCTGTGGTTGAGGGGATTCGGTGC	446–476
DAdV-3-100k-R4	CTCAGCCTGTGGTTGAGGGGATTCGGTGGGC	444–474
DAdV-3-100k-crRNA	CACCUCAGACCAUUCCGACACUG	312–334

### 2.4 Establishment and optimization of the RAA amplification system

The RAA reaction mixture was prepared according to the manufacturer’s instructions, with a total volume of 20 μL: 10 μL of Rehydration Buffer (2×), 1 μL each of forward and reverse primers (10 μM), 2 μL of DNA template, 2 μL of Starter, and 4 μL of ddH₂O. The reaction was incubated in a constant-temperature water bath, and parameters including primer sets, incubation temperature (37 °C, 38 °C, 39 °C, 40 °C, 41 °C, and 42 °C), and incubation time (15 min, 20 min, 25 min, 30 min, 35 min, and 40 min) were systematically evaluated. Amplification products were analyzed by 3% agarose gel electrophoresis and visualized using a gel imaging system. Optimal primers and reaction conditions were selected based on amplification results.

### 2.5 Establishment and optimization of the RAA-CRISPR/Cas12a-LFD assay

The CRISPR/Cas12a reaction mixture (20 μL) was assembled as follows: 2 μL Cas12a Reaction Buffer, 1 μL Cas12a protein (1 μM), 1 μL crRNA (1 μM), 2 μL RAA amplicon, 0.25 μL Cas12a reporter (4 μM), and 13.75 μL ddH₂O. An orthogonal experimental design was used to test Cas12a protein concentrations (50 nM, 150 nM, 250 nM, 350 nM, 450 nM) and crRNA concentrations (50 nM, 150 nM, 250 nM, 350 nM, 450 nM) using DAdV-3 RAA products as the template. The reaction mixture was incubated in a water bath at 37 °C (for 15 min, 30 min, 45 min, 60 min, and 75 min). After incubation, 2 μL of product was mixed with 78 μL dilution buffer, and Lateral flow dipstick were inserted into the mixture. Results were recorded within 5 min to identify the optimal Cas12a/crRNA concentration combination and the reaction time.

### 2.6 Specificity evaluation of the RAA-CRISPR/Cas12a-LFD assay

Using the optimized RAA conditions, nucleic acids from DAdV-3, avian influenza virus (AIV), Newcastle disease virus (NDV), Duck Tembusu virus (DTMUV), Duck plague virus (DPV), Muscovy duck parvovirus (MDPV), Duck hepatitis A virus type 1 (DHAV-1), and Muscovy duck reovirus (DRV) were amplified. The resulting RAA products served as templates for CRISPR/Cas12a detection, with ddH₂O as the negative control. The specificity of the assay was assessed by observing signal development on Lateral flow dipstick within 5 min.

### 2.7 Sensitivity evaluation of the RAA-CRISPR/Cas12a-LFD assay

The DAdV-3100 K plasmid was serially diluted ten-fold from 2.87 × 10^4^ to 2.87 copies/μL to generate five dilution levels. Each dilution was tested using the optimized RAA-CRISPR/Cas12a assay, with a ddH₂O control. Sensitivity was determined by observing test line signals on Lateral flow dipstick within 5 min.

### 2.8 Reproducibility assessment of the RAA-CRISPR/Cas12a-LFD assay

Two concentration levels (2.87 × 10^4^ copies/μL and 2.87 × 10^2^ copies/μL) from the dilution series were tested in triplicate using the optimized assay. ddH₂O was included as the negative control. Intra- and inter-assay reproducibility were evaluated based on consistent Lateral flow dipstick readouts within 5 min.

### 2.9 Clinical sample detection

To validate the established RAA-CRISPR/Cas12a-LFD detection method, a total of 84 clinical samples collected from Fujian were tested, including Muscovy ducks (45 samples), Cheery valley ducks (23 samples), and Ma ducks (16 samples). Nucleic acids were extracted from the tissue samples using a rapid nucleic acid extraction kit. The samples were detected using both the established RAA-CRISPR/Cas12a-LFD method and the real-time qPCR method previously established by us (Wan et al., 2018).

## 3 Results

### 3.1 Establishment and optimization of the RAA amplification system

**Figure 1:**
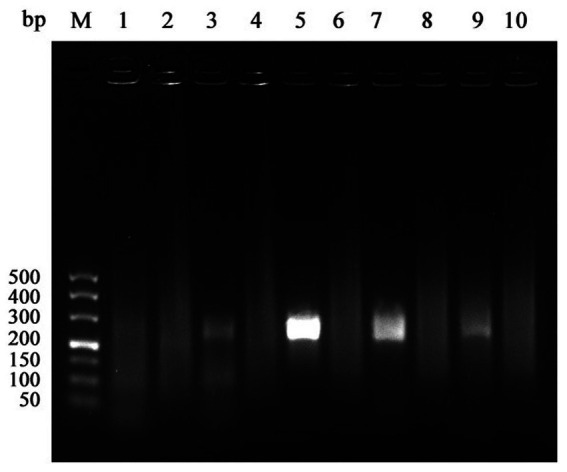
Optimization results of primer pairs in RAA reaction system. (M) 500 bp Maker; (1) DAdV-3-100 k-F1/R1; (2) DAdV-3-100 k-F1/R1 negative control; (3) DAdV-3-100 k-F2/R2; (4) DAdV-3-100 k-F2/R2 negative control; (5) DAdV-3-100 k-F2/R3; (6) DAdV-3-100 k-F2/R3 negative control; (7) DAdV-3-100 k-F3/R1; (8) DAdV-3-100 k-F3/R1 negative control; (9) DAdV-3-100 k-F4/R4; (10) DAdV-3-100 k-F4/R4 negative control.

Five primer pairs targeting the DAdV-3100 K gene were designed and screened. Agarose gel electrophoresis showed that the primer pair DAdV-3-100 k-F2 and DAdV-3-100 k-R3 (referred to as primer set 3, Table 1) produced a distinct amplification band (Figure 1). Accordingly, this primer pair was selected as the optimal RAA primer set for DAdV-3 amplification.

**Figure 2:**
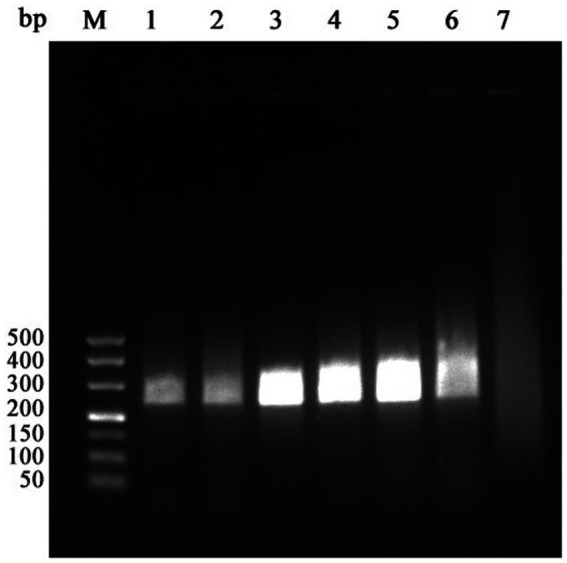
Optimization results of RAA reaction temperature (M: 500 bp Maker; 1: 37 °C; 2: 38 °C; 3: 39 °C; 4: 40 °C; 5: 41 °C; 6: 42 °C; 7: Negative control).

For temperature optimization, reactions were performed at 37 °C, 38 °C, 39 °C, 40 °C, 41 °C, and 42 °C. Specific amplification products were obtained at 39 °C, 40 °C, and 41 °C. Considering energy consumption and heating efficiency, 39 °C was selected as the optimal reaction temperature (Figure 2).

**Figure 3:**
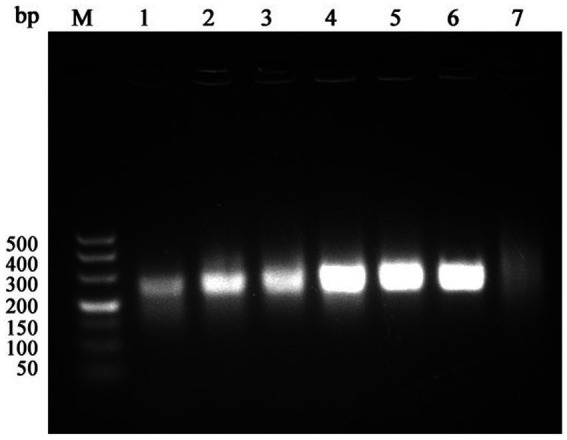
Optimization results of RAA reaction time (M: 500 bp Maker; 1: 15 min; 2: 20 min; 3: 25 min; 4: 30 min; 5: 35 min; 6: 40 min; 7: Negative control).

For time optimization, reactions were conducted for 15 min, 20 min, 25 min, 30 min, 35 min, and 40 min (Figure 3), amplification was observed at all time points, and band intensity plateaued at 30 min. Therefore, 30 min was designated as the optimal reaction time to ensure rapid amplification without loss of signal intensity.

### 3.2 RAA-CRISPR/Cas12a-LFD interpretation criteria

The CRISPR reaction system was prepared following the instructions for the Cas12a reaction reagent. The reaction product was mixed with a competitive nucleic acid lateral flow dipstick, and results were determined by observing the bands on the strip. The intact reporter molecule was labeled with FAM and biotin at both ends. The test line (T line) of the dipstick was coated with anti-FAM antibody, and the control line (C line) was coated with anti-mouse antibody. After sample application, the complex formed by the binding of biotin to colloidal gold-labeled mouse antibody migrated to the T line, where the anti-FAM antibody captured the intact reporter, producing a visible band. Meanwhile, the anti-mouse antibody on the C line captured the colloidal gold-labeled antibody, generating the control band. The appearance of both T and C lines indicated a negative result. When the target nucleic acid was present, the trans-cleavage activity of Cas12a was activated, resulting in cleavage of the single-stranded DNA reporter. The cleaved reporter could not be captured at the T line, leading to no band formation. The unbound colloidal gold complex continued to migrate to the C line, where it was captured by the anti-mouse antibody, producing a visible band. The appearance of only the C line indicated a positive result.

Therefore, the interpretation of results for the RAA-CRISPR/Cas12a-LFD reaction system is as follows: Bands appearing on both the T line and C line indicate a negative result; no band on the T line while a band appears on the C line indicates a positive result; a band on the T line with no band on the C line, or no red bands on either the T line or C line, indicates an invalid test result.

### 3.3 Optimization results of the RAA-CRISPR/Cas12a-LFS

**Figure 4:**
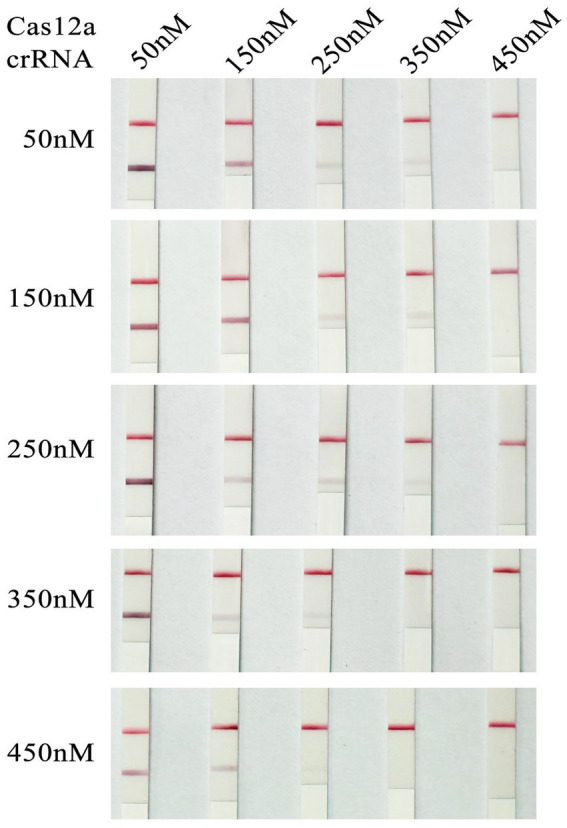
Optimization results of concentrations of Cas12a and crRNA (Cas12a 350 *n*; CrRNA 350 nM).

Experiments were conducted by setting concentration gradients for the Cas12a protein and crRNA. The results (Figure 4) indicated that when the concentration of the Cas12a protein in the system was 350 nM and the crRNA concentration was 350 nM, the test dipstick results began to show stable positivity. Consequently, the optimal reaction system for RAA-CRISPR/Cas12a was determined to be 20 μL, consisting of: 2 μL of Cas12a Reaction Buffer (10×), 1.4 μL of Cas12a Protein (5 μM), 0.25 μL of Cas12a Reporter (4 μM), 1.4 μL of crRNA (5 μM), 2 μL of RAA amplification product, and 12.95 μL of ddH₂O. In all subsequent experiments, Cas12a at a concentration of 350 nM and crRNA at a concentration of 350 nM were used.

**Figure 5:**
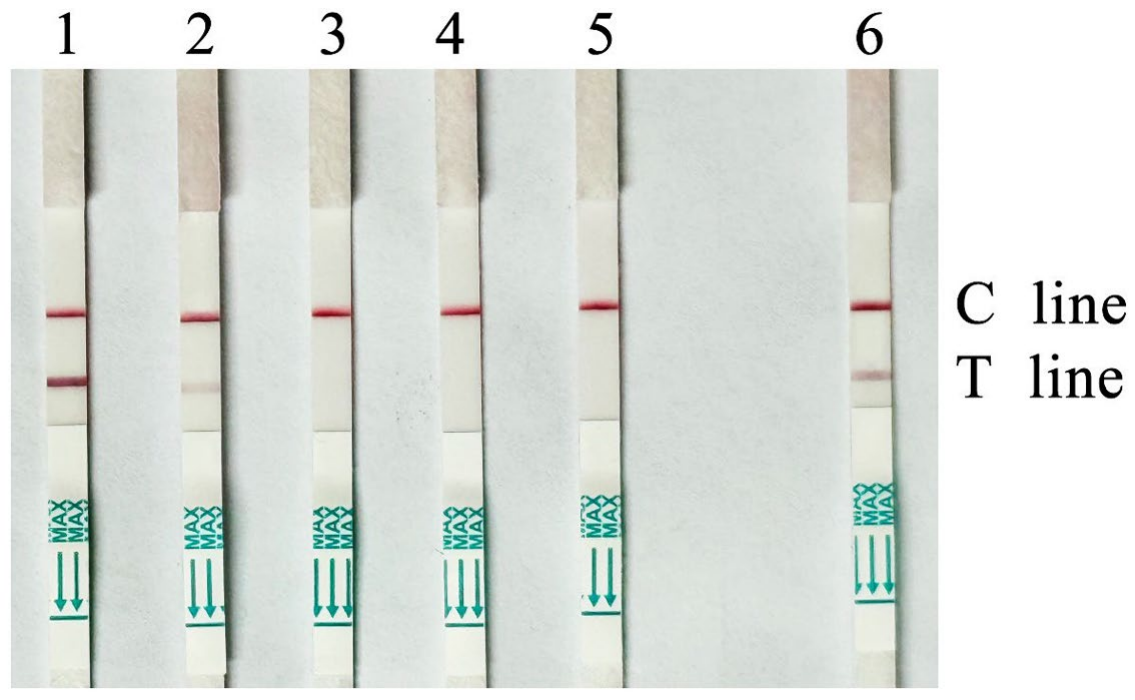
Optimization results of RAA-CRISPR/Cas12a-LFD reaction time (1: 15 min; 2: 30 min; 3: 45 min; 4: 60 min; 5: 75 min; 6: Negative control).

Using the optimal concentrations of Cas12a protein and crRNA, a reaction time optimization experiment was performed with reaction times set at 15 min, 30 min, 45 min, 60 min, and 75 min. The results are shown in Figure 5. Positive results were obtained at both 45 min and 55 min. To meet the requirement for rapid detection, the optimal reaction time was determined to be 45 min.

### 3.4 Results of specificity test

**Figure 6:**
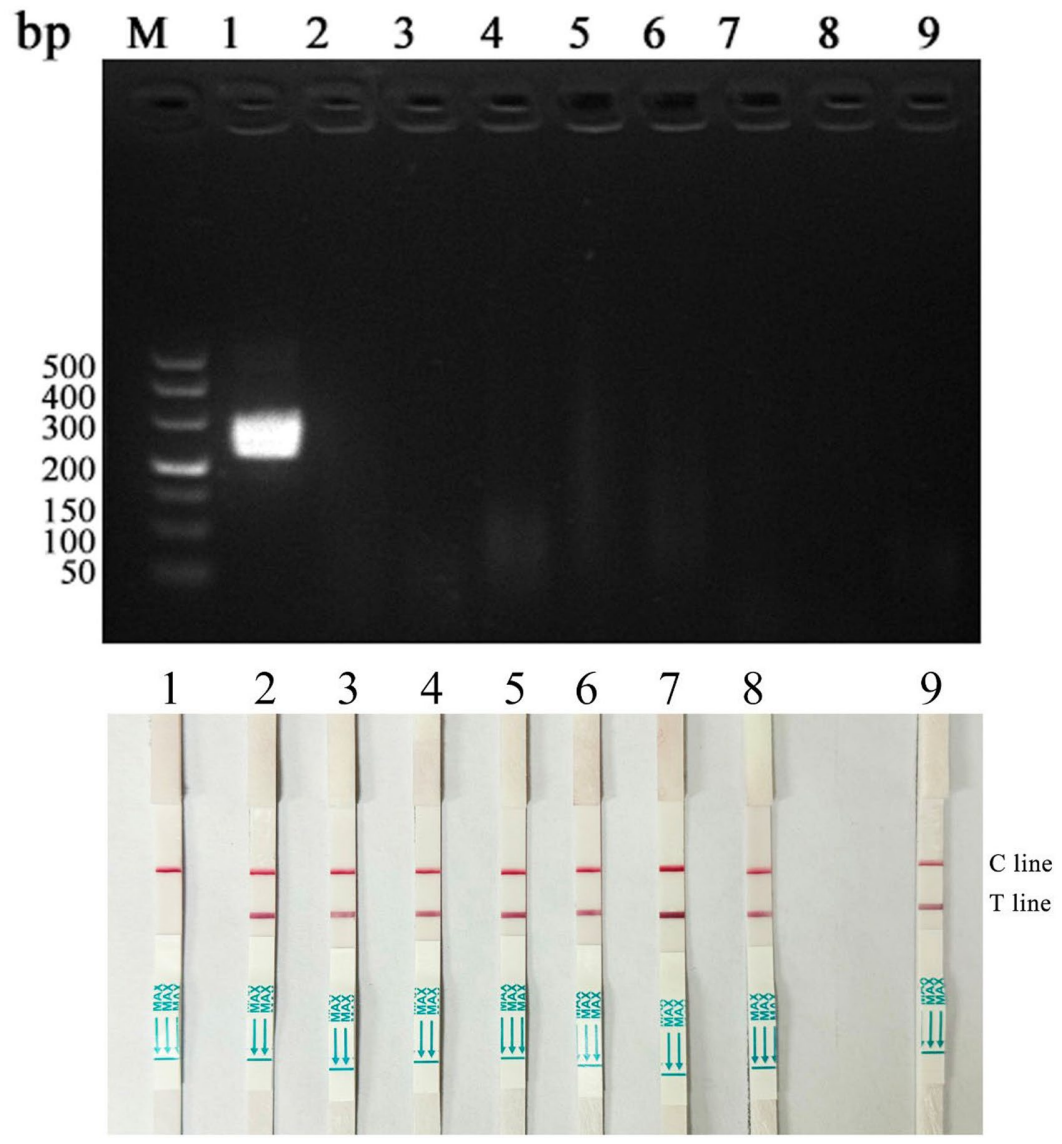
Specificity test of RAA-CRISPR/Cas12a-LFD assay (M: 500 bp Maker; 1: DAdV-3; 2: AIV; 3: NDV; 4: DTMUV; 5: DPV; 6: MDPV; 7: DHAV-1; 8: DRV; 9: Negative control).

Specificity tests were conducted using the optimized detection method. The results (Figure 6) indicated that this method could specifically amplify only DAdV-3, with no amplification observed for other duck-origin viruses such as AIV, NDV, DTMUV, DPV, MDPV, DHAV-1, and DRV. This demonstrates the high specificity of the established method.

### 3.5 Results of sensitivity test

**Figure 7:**
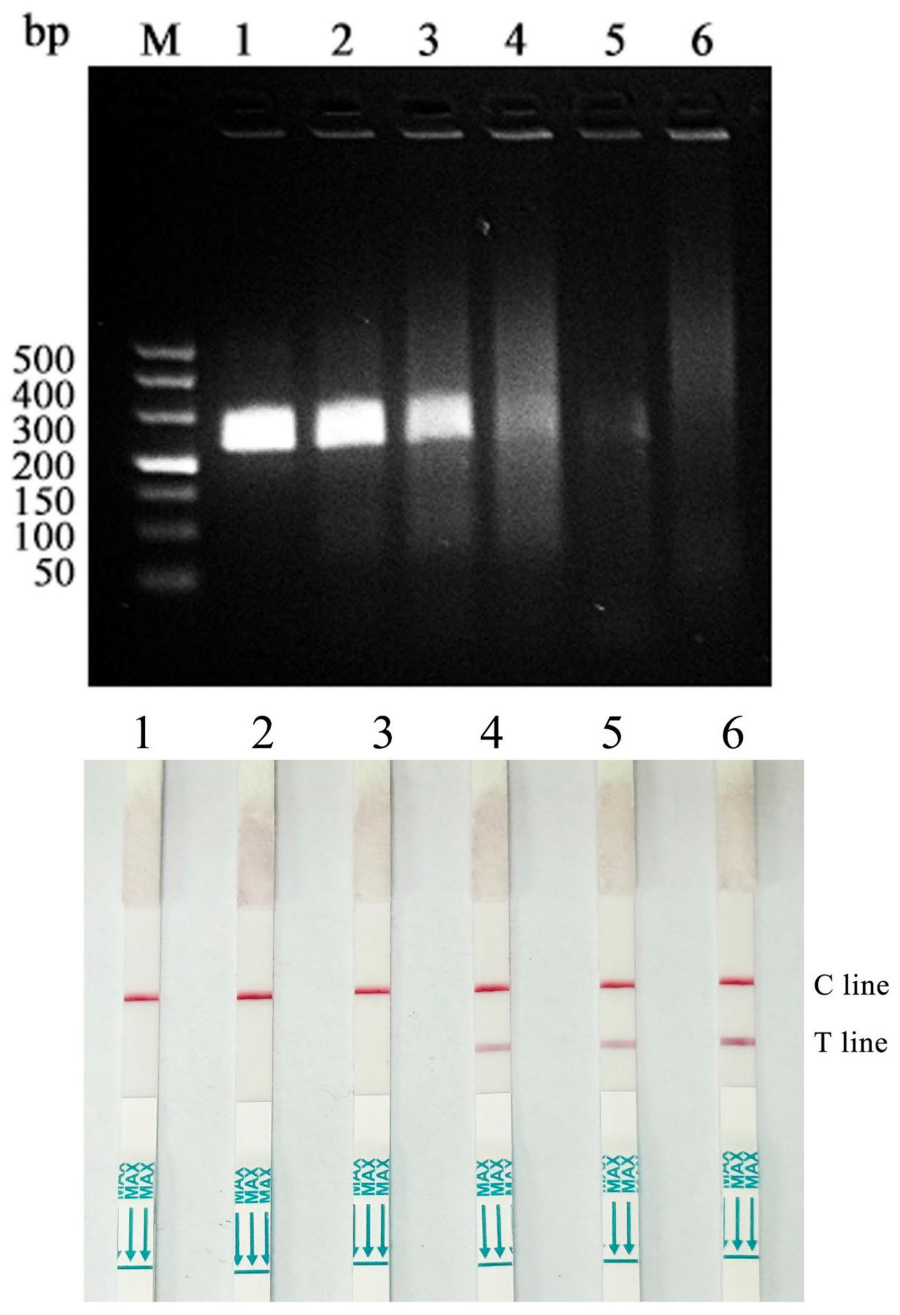
Sensitivity test of RAA-CRISPR/Cas12a-LFD assay (M: 500 bp Maker; 1: 2.87 × 10^4^ copies/μL; 2: 2.87 × 10^3^ copies/μL; 3: 2.87 × 10^2^ copies/μL; 4: 28.7 copies/μL; 5: 2.87 copies/μL; 6: Negative control).

The DAdV-3 standard plasmid was serially diluted 10-fold to create five gradients ranging from 2.87 × 10^4^ to 2.87 copies/μL. These dilutions were used as templates for the RAA-CRISPR/Cas12a-LFD reaction. The minimum copy number detectable by RAA was 2.87 × 10^2^ copies/μL, and the detection limit of the RAA-CRISPR/Cas12a combined with the test dipstick was also 2.87 × 10^2^ copies/μL (Figure 7).

### 3.6 Results of repeatability test

**Figure 8:**
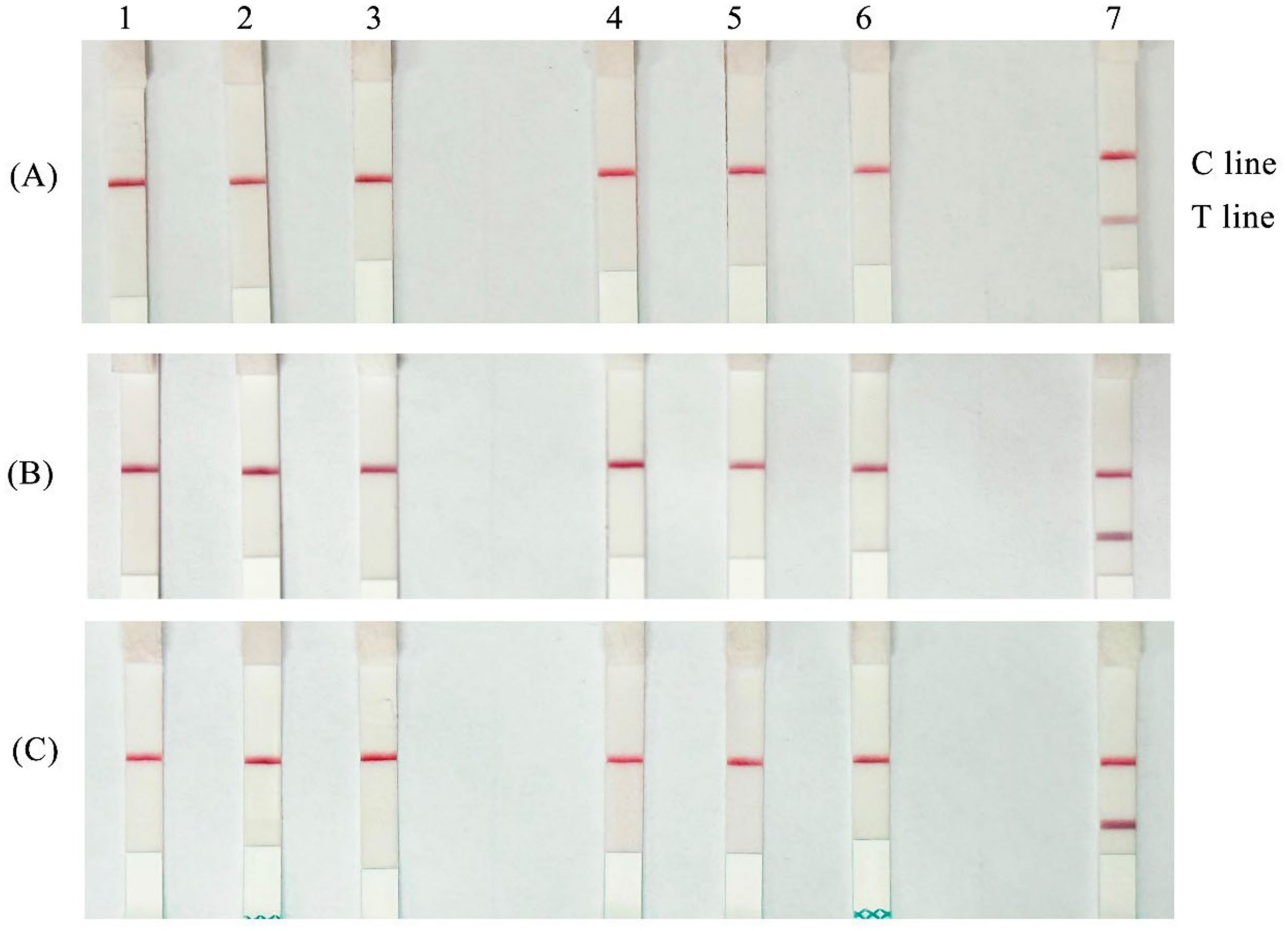
Repeatability test of RAA-CRISPR/Cas12a-LFD assay. **(A)** First repeatability test; **(B)** Second repeatability test; **(C)** Third repeatability test (1–3: 2.87 × 10^4^ copies/μL; 4–6: 2.87 × 10^2^ copies/μL; 7: negative controls).

RAA-CRISPR/Cas12a-LFD reactions were performed using positive nucleic acids at two concentrations from a gradient dilution, specifically 2.87 × 10^4^ copies/μL and 2.87 × 10^2^ copies/μL. Each sample was tested in triplicate, and all three test dipstick results were positive (Figure 8), indicating good inter-assay and intra-assay reproducibility.

### 3.7 Detection results of clinical samples

Simultaneous testing of 84 clinical samples showed that, using the RAA-CRISPR/Cas12a-LFD, 11 samples from Muscovy ducks were positive for DAdV-3, with a positive rate of 24.44% (11/45). In comparison, the qPCR method detected 12 positive samples from Muscovy ducks, yielding a positive rate of 26.67% (12/45). No positive signals were observed in Cheery valley ducks or Ma ducks (Table 2).

**Table 2:** Detection of DAdV-3 in clinical samples.

Species	Number	RAA-CRISPR/Cas12a-LFD		qPCR	
		Positive samples	Positive ratio (%)	Positive samples	Positive ratio (%)
Muscovy ducks	45	11	24.44	12	26.67
Cheery valley ducks	23	0	0	0	0
Ma ducks	16	0	0	0	0

## 4 Discussion

Clinically, DAdV-3 predominantly infects Muscovy ducks, with reported morbidity and mortality rates ranging from 40–55% and 35–43%, respectively, which aligns with previous findings particularly among young birds where infection often leads to acute death, imposing substantial economic losses on the duck farming industry in China (Shi et al., 2022). Initial clinical signs include depression, anorexia, and white, loose feces, progressing to lethargy and somnolence as immune competence declines. Necropsy commonly reveals pronounced lesions in the liver, kidney, and heart, with the liver exhibiting the most severe pathology, and histopathology typically showing extensive inflammatory cell infiltration in hepatic tissue (Tan et al., 2024). Despite the significant impact of DAdV-3, there are currently no commercially available diagnostic kits or vaccines for clinical use, underscoring the necessity of rapid, sensitive, specific, and visual detection methods for early diagnosis and disease management.

Common detection methods for DAdV-3 include PCR, qPCR, and LAMP. However, both PCR and qPCR involve dozens of amplification cycles, and each cycle requires three steps—denaturation, annealing, and extension—with precise control of temperature and time at each step. Compared with the approximately 1 h required for the RAA-CRISPR/Cas12a assay, qPCR typically requires about 1.5 h, while conventional PCR may take more than 2 h to complete. In addition, PCR and qPCR require relatively strict experimental conditions, often relying on well-trained personnel and expensive thermal cycling instruments, whereas the RAA (Recombinase-Aided Amplification) reaction can be performed using only a simple heating device, making PCR-based methods less suitable for on-site rapid diagnosis in resource-limited settings. Although LAMP eliminates the need for complex thermal cycling equipment, it requires 4–6 specially designed primers and a relatively high reaction temperature (typically around 65 °C). In contrast, the RAA reaction requires only one pair of primers, and the reaction temperature (37–42 °C) is close to physiological temperature, which further reduces the energy consumption and performance requirements of the heating device, making it more suitable for rapid field detection.

While standalone RAA provides rapid amplification, it is susceptible to false positives due to primer dimer formation and nonspecific amplification. In contrast, coupling RAA with a CRISPR-Cas system introduces an additional specificity filter: CRISPR crRNA specifically binds to the target amplicon, and only perfectly matched RAA products activate Cas-mediated cleavage, yielding dual specificity via primer hybridization and crRNA targeting. The isothermal nature of RAA eliminates reliance on thermal cyclers, and CRISPR-mediated signal amplification does not require additional amplification steps, combining speed and portability. When integrated with Lateral flow dipstick for readout, this approach enables visual detection without instrumentation. With the rapid advancement of CRISPR technologies, platforms such as Cas12a-based HOLMES and DETECTR, and Cas13a-based SHERLOCK have demonstrated mature applications in nucleic acid detection, providing a solid technological foundation for RAA-CRISPR combined systems (Chen et al., 2018; Li et al., 2018; Gootenberg et al., 2017).

RAA-CRISPR/Cas12a assays have been widely developed and optimized for diverse pathogens, including pigeon paramyxovirus, porcine rotavirus, and feline parvovirus, demonstrating broad applicability due to simplicity, sensitivity, and reliability (Liang et al., 2024; Huang et al., 2024; Chen H. et al., 2025). Unlike traditional laboratory methods, RAA-CRISPR/Cas12a-LFD does not require expensive temperature-control systems or electrophoresis, and results are visualized by lateral flow dipstick, making it suitable for point-of-care testing in resource-limited settings. To date, no RAA-CRISPR/Cas12a-LFD assay targeting the nonstructural 100 K gene of DAdV-3 has been reported. In the present study, the optimized RAA-CRISPR/Cas12a-LFD assay operates at 39 °C for 30 min for RAA, followed by a 37 °C CRISPR reaction with Cas12a and crRNA each at 350 nM for 45 min, allowing completion of the entire workflow within 1 h. This method exhibited high specificity for DAdV-3, with no cross-reactivity observed with other common duck-origin viruses, including AIV, NDV, DTMUV, DPV, MDPV, DHAV-1, and DRV. Two different plasmid concentrations were tested in three independent replicates, and all results were positive, indicating that the method has good reproducibility and stability. The detection limit for the DAdV-3 positive plasmid was 2.87 × 10^2^ copies/μL. However, it should be noted that this sensitivity was determined based on standard plasmid templates. Because plasmid DNA extraction is highly efficient and relatively simple, the sensitivity obtained under ideal conditions may differ from that achieved with real clinical samples, and the actual detection performance in field samples may be lower than that measured using purified plasmids. To further evaluate the reliability of this method, a total of 84 clinical samples from Fujian were tested in parallel using the established RAA-CRISPR/Cas12a-LFD method and a highly sensitive qPCR method. The results showed that the positive detection rate of the RAA-CRISPR/Cas12a-LFD in clinical samples from Muscovy ducks was 24.44% (11/45), comparable to the 26.67% (12/45) obtained by qPCR, with a concordance rate of 91.67% between the two methods, demonstrating that the RAA-CRISPR/Cas12a-LFD can also meet the requirements for routine clinical sample detection. No positive results were observed in Cheery valley ducks or Ma ducks, a phenomenon that may be attributed to factors such as viral host specificity, sampling regions, and breeding management practices. Further studies with expanded sample sizes and broader sampling regions are warranted to clarify the infection status of DAdV-3 in different duck breeds.

## 5 Conclusion

In summary, the RAA-CRISPR/Cas12a-LFD assay developed in this study provides a rapid, highly sensitive, specific, and user-friendly field diagnostic platform for DAdV-3. This method offers technical support for epidemiological surveillance, early diagnosis, and disease control in duck production systems.

## Data Availability

The raw data supporting the conclusions of this article will be made available by the authors, without undue reservation.

## Footnotes

1
https://www.ncbi.nlm.nih.gov/
